# Dietary Fiber and Intestinal Health of Monogastric Animals

**DOI:** 10.3389/fvets.2019.00048

**Published:** 2019-03-04

**Authors:** Rajesh Jha, Janelle M. Fouhse, Utsav P. Tiwari, Linge Li, Benjamin P. Willing

**Affiliations:** ^1^Department of Human Nutrition, Food and Animal Sciences, University of Hawaii at Manoa, Honolulu, HI, United States; ^2^Department of Agricultural Food and Nutritional Science, University of Alberta, Edmonton, AB, Canada

**Keywords:** dietary fiber, gut health, gut microbiota, immunity, nutritional strategy, pig, poultry, gene expression

## Abstract

Animal performance, feed efficiency, and overall health are heavily dependent on gut health. Changes in animal production systems and feed regulations away from the use of antibiotic growth promoters (AGP) have necessitated the identification of strategies to optimize gut health in novel and effective ways. Among alternatives to AGP, the inclusion of dietary fibers (DF) in monogastric diets has been attempted with some success. Alternative feedstuffs and coproducts are typically rich in fiber and can be used in the diets to reduce feed costs and optimize gut health. DF are naturally occurring compounds with a diverse composition and are present in all plant-based feedstuffs. DF stimulate the growth of health-promoting gut bacteria, are fermented in the distal small intestine and large intestine to short-chain fatty acids and have beneficial effects on the immune system. Maternal DF supplementation is one novel strategy suggested to have a beneficial programming effect on the microbial and immune development of their offspring. One mechanism by which DF improves gut health is through maintenance of an anaerobic intestinal environment that subsequently prevents facultative anaerobic pathogens from flourishing. Studies with pigs and poultry have shown that fermentation characteristics and their beneficial effects on gut health vary widely based on type, form, and the physico-chemical properties of the DF. Therefore, it is important to have information on the different types of DF and their role in optimizing gut health. This review will provide information and updates on different types of DF used in monogastric nutrition and its contribution to gut health including microbiology, fermentation characteristics, and innate and adaptive immune responses.

## Introduction

Although dietary fiber (DF) is abundantly present in common feedstuffs, its concentration in monogastric animal diets has increased proportionally with the increased incorporation of coproducts. It is well-known that DF can contribute nutritional value to animals, directly by providing energy (1, 2) and indirectly by improving gut health and immune function (3–6). Yet, DF has historically been considered as an antinutritional factor due to its negative impacts on nutrient utilization (4, 7). However, DF has recently gained special attention due to its functional value in improving gut health of monogastric animals (8). Maintaining or improving gut health is essential to enhance feed efficiency, promote growth performance, and maintain the overall health of monogastric animals. Antibiotic growth promoters (AGP) have been used in feeding programs for over 60 years to maintain or promote gut health and improve growth performance of production animals. However, due to potential public health risks, use of AGP have been banned or tightly regulated in several countries. To overcome the negative impacts of AGP regulation and ban on health and productivity of animals, several alternatives have been proposed and tested; with DF being considered to be one of the effective alternatives to AGP (8).

DF are naturally occurring compounds with a diverse composition and are present in all plant-based feedstuffs including cereals, tubers, and agro-industrial byproducts (8–10). Despite some adverse effects on nutrient and energy digestibility, there is growing interest for including DF in monogastric animal diets due to its potential beneficial effects on the gut health, welfare, and the environment (11). DF escapes digestion by host endogenous enzymes in the proximal small intestine and is utilized by the residing microbial population as a fermentative substrate in the distal small intestine and large intestine. Microbial fermentation of DF produces metabolites including short-chain fatty acids (SCFA), which in turn, promotes the growth of beneficial gut bacteria, supports intestinal integrity, and proper immune function. Studies with pigs and poultry have shown that fermentation characteristics and their beneficial effects on gut health vary widely based on type, form, and the physico-chemical properties of the DF (8) as well as the matrix in which it lies (12). Therefore, it is important to have information on the different types of DF and their specific roles in optimizing gut health of monogastric animals.

This paper has reviewed different types of DF used in monogastric animals (primarily pigs and poultry) and their role in modulating intestinal health. To gain a better understanding of this topic, we have discussed the effects of DF on pigs and poultry nutrient utilization and its fermentation characteristics. For further comprehension, we have highlighted the influence of DF on intestinal mucosa and histomorphology, microbial profiles of both host animals and progeny, and innate and adaptive immune response. Finally, we have emphasized the effect of DF on intestinal disorders and diseases.

## Dietary Fiber

Dietary fiber can be defined in many ways; most commonly being based on the chemical composition and the physiological functions. Based on chemical composition, DF is the sum of non-starch polysaccharides (NSP) and lignin. From a nutritionist's point of view, it can be simply defined as carbohydrates that are indigestible by endogenous enzymes. Common feed ingredients rich in fiber are cereals like barley, wheat, oats, and other coproducts like distillers dried grains with solubles, canola meal, and wheat millrun. Generally, DF includes cell wall components cellulose, hemicellulose, and other structural and non-structural compounds resistant starch (RS), inulin, chitin, pectin, β-glucan, and oligosaccharides. The utilization of DF in pig and poultry diets depends on the fiber content, the degree of microbial fermentation in the large intestine, the extent of absorption, and other factors (8, 13). Soluble fiber sources are rapidly fermented by resident microbes in the distal small intestine and large intestine, increase digesta viscosity, reduce digesta passage rate through the intestine, and can decrease feed intake due to increased satiety. On the other hand, insoluble fiber passes through the intestine undigested, increases passage rate and fecal bulking; however, monogastric species have a limited capacity to ferment insoluble fiber as they lack specific microbial species (4, 14). Therefore, it is essential to understand the components of DF and its nutritional and physiological effects in animals before incorporating it into monogastric diets. For details on the composition of DF, its sources and utilization in different parts of the gastro-intestinal tract (GIT), readers are referred to Jha and Berrocoso (8), which provides an extensive updated review on these topics.

## The Concept of Intestinal Health

**Figure 1:**
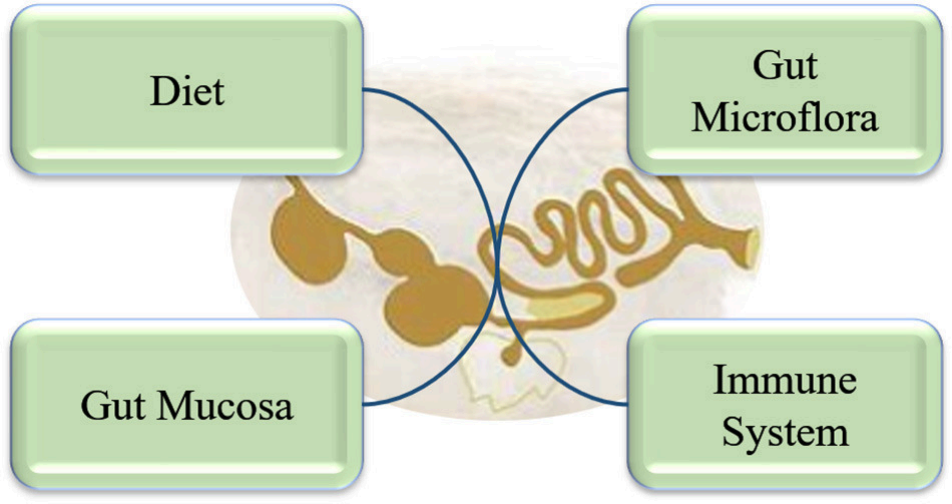
Components of gut health in a holistic approach.

The GIT is the largest group of organs in the body. It is not only the site of digestion and absorption of dietary nutrients but provides protection against pathogens and toxins. Moreover, it hosts a large population of microbiota and immune cells. Thus, a healthy intestinal tract is of utmost importance for overall sound health and improved productivity of animals. However, the definition of “intestinal health” or “gut health” is not yet clearly defined, despite it having been a focus of major research efforts in the last few decades. Conway (15) proposed that gut health is the function of three major components: the diet, the mucosa, and the commensal microbiota. Later, Montagne et al. (16) elaborated that it includes a diet that would provide sufficient nutrients, mucosa that maintains the gut integrity, and a microbial community that maintains a balanced, healthy environment. Since the GIT of pigs and poultry contains about 70% of total body immune cells, it should be included in the definition of “intestinal health.” Thus, we suggest that intestinal health should be considered in a holistic way including the diet, mucosa, microbiome, and immune system (Figure 1). The GIT of pigs and poultry consists of hemopoietic cells (macrophages, dendritic cells, and T-cells), non-hemopoietic cells (epithelia, Paneth cells, and goblet cells), and the microbiome (bacteria, archaea, protists, fungi, and viruses) all of which contribute to gut health. The innate and adaptive immune systems constantly communicate with the microbiome to maintain homeostasis. Any imbalance in the immune system or the microbiome can lead to dysbiosis, resulting in increased susceptibility to various diseases (17). The intestinal mucosa is composed of the epithelium, the gut-associated lymphoid tissue (GALT), and the mucus overlying the epithelium. The intestinal mucus, host epithelial cells, GALT, and microbiome interact with each other forming a fragile and dynamic equilibrium, which is critically important for efficient functioning and absorption capacity of the digestive system. The physical (epithelial cells, intercellular tight junction, and mucus) and chemical (acidity, proteolytic enzymes, lysozymes, and antibacterial proteins) barriers play an important role in maintaining gut barrier function and preventing the microbial population from translocating and causing systemic immune activation. Besides acting as a physical barrier, the epithelial cells also secrete cytokines and chemokines that regulate chemotaxis of immune cells. Paneth cells located at the base of crypts of many vertebrate species, including poultry. It contains defensin rich granules that are released in response to bacterial-induced inflammation (not during protozoal or fungal infection) via exocytosis (18). Three mucosal barrier factors help to maintain and restore the mucosal integrity of intestine; diamine oxidase, trefoil factor, and transforming growth factor-α. Occludin, claudin, and zona occludens-1 are the three tight junction proteins that maintain the paracellular barrier (19). Goblet cells in the GIT produce mucin, which also plays an important role in maintaining gut barrier function. Mucin production can be increased several bacteria, including *Lactobacillus* (20), which can help to improve the gut barrier as pathogenic microbes are impeded by the dense mucous layer. However, optimal gut health is not characterized by complete absence of pathogenic microbiota, rather an intestinal microbiome with a high microbial and functional diversity.

## DF and Nutrient Utilization

The significant fraction of NSP in any cereals fed to pigs or poultry consists of arabinoxylan, followed by cellulose, and mixed linked β-glucan (8, 21). Cellulose is a polysaccharide consisting of chains of glucose molecules. It differs from starch in the orientation of the glycosidic bonds. While starch has α-glycosidic bonds, those in cellulose are in a β-orientation. Lignins are cross-linked phenol polymers and are present in a more significant proportion in rye than in wheat and oat, with a concentration in bran higher than in whole grain (21). Among the commonly used cereals in the diets of pigs and poultry, the concentration of β-glucans is the highest in oat (4%), intermediate in wheat and rye (0.7–1.7%), and lowest in corn (0.1%) (21). The structure of the cell wall of cereal grains is complex, and their composition and properties vary depending upon the location of tissues. The kernel of the cell wall consists of xylans, cellulose, and a significant amount of lignin. This layer is thick and hydrophobic. On the other hand, endosperm (aleurone layer) is thin and hydrophilic and consists of mainly two polysaccharides, arabinoxylans, and β-glucan (22). NSP present in cell walls, along with lignin, are not digested by endogenous enzymes but can influence digestion and absorption by encapsulating nutrients and by increasing digesta viscosity (23). The concentration of DF in brans are generally far greater than in whole grains. Most brans contain a higher amount of insoluble fiber than cereal grains with the exception of oat bran which is more soluble as it contains a larger aleurone and sub-aleurone layer and higher amounts of β-glucan (24). The aleurone layer in wheat contains a large amount of arbinoxylan as well as phenolic phytochemicals. The aleurone layer is a part of the endosperm and contains higher amounts of insoluble polysaccharides than the remaining endosperm layers (21). The aleurone and pericarp also contain increased amounts of ferulic acids than in the starchy endosperm layer (25). Ferulic acid is the most abundant phenolic acid present in most cereals and wheat and rye brans, which are esterified to arabinoxylans. The physicochemical properties of DF are affected by the crosslinking of diferulates with lignin, with insoluble DF possessing 100 times higher diferulates than soluble DF (26).

Amount of DF and nutrient utilization are inversely proportional to each other. Increases in the amount of DF reduce growth performance of monogastric animals. However, the inclusion of NSPase or the fiber degrading enzymes has been found to be one of the best methods of eliminating the negative effects of DF on growth depending on the type and structure of fiber present in the ingredients used (23, 27, 28). Structural component, orientation, substitution, presence of functional group; all has a role to play in determining the effect of DF in gut immunity. The immunomodulating effect of DF has been reported to have overall health benefits to host animals (23) describing its potential to be used as an alternative to AGP (27). Increased regulations and the banning of sub-therapeutic antibiotics in monogastric diets have led nutritionists to look for alternative strategies to maintain animal growth performance. Therefore, dietary inclusion of oligosaccharides and soluble fiber is one potential alternative strategy to help support gut health and animal performance.

## DF Fermentation and Effects

The diet of pigs consists of a considerable amount of carbohydrates, which partially escapes small intestinal digestion, and passes through to the large intestine where it is fermented by microbes. Microbial fermentation of DF results in the production of SCFA, branched chain fatty acids (BCFA), lactate, amines, indoles, phenols, and various gasses like hydrogen, carbon dioxide, and methane (11). The substrate that is being provided to microbes to ferment directs the end metabolites. In the absence of adequate DF, proteolytic fermentation can take place in the colon producing BCFA and potentially harmful metabolites like ammonia indoles, and phenols. Ammonia is produced from the deamination of amino acids and hydrolysis of urea whereas phenols are produced due to carboxylation of amino acids. Hence, the composition of SCFA produced in the gut can be manipulated by changing the substrate that reaches the colon (4, 5, 29).

Starch digestion in pigs is more desirable than its fermentation to SCFA because starch digestion products are more efficient sources of energy (30, 31). The SCFA are thought to provide up to 15% of the maintenance energy requirement of growing pigs and 30% in gestating sows (1). However, an increase in the concentration of SCFA, more specifically of butyrate, can improve the gut mucosal health as well as the immune system of pigs. Energy provided by butyrate to the host is vital to maintaining the gut ecosystem as well as the health of pigs. In the absence of fermentable carbohydrates as an energy source, microbial fermentation shifts toward amino acids and utilize carbon skeleton from amino acids as energy source, and the resulting metabolite ammonia is absorbed and disposed of in the form of urea (11). On the other hand, in the presence of energy from fermentable carbohydrates, ammonia is removed as microbial biomass (32), i.e., the resident microbes in the large intestine retain more nitrogen for their growth.

**Figure 2:**
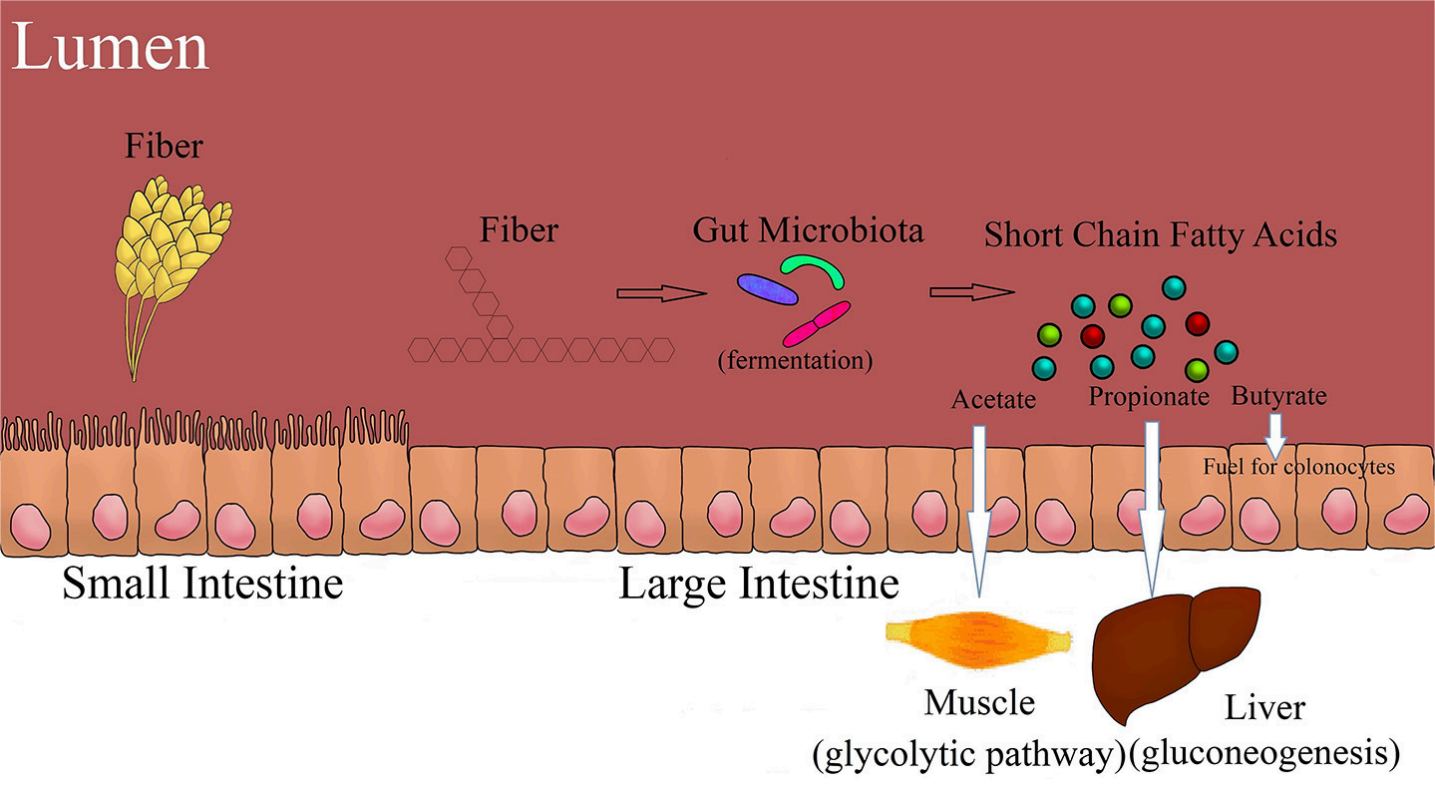
Fiber fermentation and its primary utilization pathways.

The most abundant end product of fermentation in the proximal GIT is acetate, which contributes to more than 90% of total SCFA produced. However, conditions change in the distal GIT, where the concentration of lactate decreases and the concentration of SCFA increases with a ratio of approximately 60% acetate, 25% propionate, and 15% butyrate. Degradation of DF is highest in the proximal colon, and so is the production of lactic acid and SCFA. However, the progressive decrease in the flow of digesta toward the distal colon changes the fermentation metabolite and bacterial profile (4, 6). Modification in the structure of DF due to cross-linking, transglycosylation, or esterification prevents hydrolysis of starch both by the host and bacterial enzymes. Most of the SCFA (more than 90%) absorption occurs in the anionic dissociated form, as they are weak acids. The SCFA produced are absorbed from the apical membrane by three primary methods; passive diffusion in lipid soluble form, anion exchange between bicarbonate and SCFA (33), and by the help of active transporters like Monocarboxylate transporter 1 (MCT1) and Sodium coupled monocarboxylate transporter 1 (SMCT1). Fermentation starts only after the DF gets depolymerized by microbial hydrolytic enzymes. The faster the rate of depolymerization of a substrate, the faster the carbohydrates will be available for fermentation by the bacteria. The DF which are heavily branched provide a larger surface area for enzymes to act on and are more rapidly fermented (30). On the other hand, degradation of linear polymers or high amylose starch is slowly fermented as their degradation yields larger fragments (larger oligomers), which are further utilized by bacteria and produce metabolites like SCFA and gases. The major fermentation metabolites and its primary utilization pathway are summarized in the Figure 2.

The solubility of DF also affects SCFA production, as insoluble DF are less fermentable compared to soluble DF because insoluble DF contains ~100-fold more ferulic acid (26). Besides SCFA production, soluble DF influences gut health by decreasing fecal bulk, delaying emptying of liquids by increasing viscosity of gastric chyme, lowering pH in the intestinal lumen as well as altering bile acid profiles (34). Soluble DF are responsible for changing viscosity of luminal digesta (23, 35). When soluble DF comes in contact with water, it absorbs it and swells, increasing the viscosity of digesta. Viscosity of DF is also affected by the molecular weight of individual DF. Structural variation, the degree of polymerization, branching, and chemical modification in the DF subsequently determine its fermentation characteristics. Solubility and viscosity of DF also affect the end product of fermentation.

## DF and Intestinal Mucosa/Histomorphology

Gut health is essential to maintain growth performance and overall health of monogastric animals. The primary role of intestinal mucosal tissue is digestion and absorption of nutrients. Feed ingredients are hydrolyzed and broken down by the host into smaller compounds; the mucosa obtains glucose from starch, amino acids, and peptides from proteins, and fatty acids and monoglycerol from lipids. The DF are fermented resulting in SCFA, which promote proliferation of the mucosal epithelium and villus height (36). The epithelial layer of mucosa regulates the exchange of nutrients to the body (16). Besides the intestinal secretions and glycoproteins produced by the brush border membrane, mucosal epithelium also greatly influences the adherence capacity and the metabolic activity of intestinal microbes. Hence, the intestinal mucosa acts as a barrier to the pathogenic bacteria and toxic compounds. Both innate and adaptive immune systems participate in the building of intestinal mucosal barrier.

The inclusion of DF often increases the endogenous losses, resulting in a perceived decrease in the digestion of energy and nutrients in monogastric animals. Therefore, DF has been recognized as “anti-nutritive” for monogastric animals. Moreover, these negative effects are more prominent to chickens and piglets than in growing and finishing pigs (37). However, moderate levels of dietary fiber may increase gut size, length, volume, and morphological structure of pigs, poultry, and other non-ruminant animals. The addition of soluble fiber to the diet of piglets generally causes an increase in the viscosity of the intestinal content, which may increase the rate of villus cell losses leading to villus atrophy (38). The villus height to crypt depth ratio is a useful criterion for estimating the likely digestive capacity of the small intestine. In growing pigs, the inclusion of 10% high fiber source in diets over 14 days caused an increased width of villi and depth of the crypts in the jejunum and ileum. The inclusion of high fiber in diets also increased the rate of cell proliferation and crypt depth in the large intestine, when compared to the same diet containing no straw (39). However, the height of villus and the depth of crypt in the gut is not immutable; it changes with the location of the small intestine. Therefore, it is critical to understand the mechanisms of nutrient absorption, and the location of specific nutrient utilization in the gut to develop the optimal feeding system to obtain the best production performance.

## Dietary Fiber and Intestinal Microbiota

### Direct Fiber Supplementation on Microbial Composition

The complex carbohydrates and plant polysaccharides indigestible by monogastric animals provide an essential fermentative substrate to the microbiome (including bacteria, fungi, protozoa, and archea) and are known to impact bacterial composition, diversity, and metabolic capabilities (40). It is likely the microbiome as whole that contributes to fiber breakdown; however, only the role bacteria play in this complex process has been well-defined. The GIT of poultry and swine are highly diverse containing over 1,000 bacterial species mainly belonging to predominant phyla Firmicutes, Bacteroidetes, and Proteobacteria (41–43). It must be taken into consideration that the nutritional and health benefits residing bacteria provide to their host is a result of the entire community and their metabolic capabilities, not the presence or absence of a single species. It is through glycoside hydrolases, polysaccharide lyases, and carbohydrate esterases that gut- associated bacterial communities are able to breakdown and ferment complex carbohydrates into SCFAs (44).

The microbial process of fiber fermentation is considerably more variable than host macronutrient digestion due to the range in fiber sources and the physicochemical properties of that fiber (i.e., solubility, viscosity, and water-holding capacity) (31). Recently recognized in humans is the substantial effect colonic transit time has on microbial composition (45). Therefore, soluble fiber has the ability to increase the viscosity of intestinal digesta and the transit time, hence increased intestinal mass. Retained digesta in intestinal lumen for longer time provides opportunity for proliferation of selective microbiota. This might be the probable mechanism which cause fiber and its type alter microbial profiles. Resistant starches are also involved in increasing the viscosity of digesta. However, RS are easily degraded to small molecular weight residue whereas DF are more resistant to depolymerization. This might be the reason for RS to have better response than DF. In weaned and growing pigs, changing passage rate and site of digestion of starch from the proximal to distal intestine through the inclusion of purified resistant starch selectively promotes bifidobacteria (46, 47) and lactobacilli as reviewed in a recent meta-analysis (48). Fermentable fiber from barley high in β-glucans also shifts the site of nutrient digestion from the small to large intestine subsequently increasing relative abundance of Firmicutes genera; *Dialister, Sharpea*, and *Ruminococcus* (49). However, increasing digesta viscosity in poultry with soluble fiber (barley β-glucans or wheat arabinoxylans) can be detrimental to growth and has shown to favor expansion of potential pathogens, *E. coli* and *Clostridium perfringens* (50–52). Viscosity caused by certain fiber results in villus cell loss as it prevents the enterocytes from reaching to the nutrients. Long term impact of such fiber inclusion results in atrophy of villi. Supplemental enzyme has shown positive response in minimizing this impact (23). The villus height to crypt depth ratio is a useful criterion for estimating the likely digestive capacity of the small intestine. In pigs, arabinoxylans enrich butyrogenic species and others commensals including *Faecalibacterium prausnitzii, Rosburia intestinalis, Blautia coccoides, Eubacterium, rectale, Bifidoabcterium, and Lactobacillis* spp. (53). A more in depth review of how specific fiber types and feed ingredients promote beneficial bacteria can be found elsewhere (8).

In comparison to swine, the literature exploring the complex interactions between gut microbiota and fiber in poultry is scarce. However, recently over 200 different non-starch polysaccharide-degrading enzymes (mainly oligosaccharide degrading enzymes vs. cellulases and endohemicellulases) were found encoded within the metagenome of broiler microbiota, suggesting poultry microbiota are capable of utilizing soluble forms of dietary fiber (41). The importance of supplying dietary fiber to the microbiota is truly demonstrated in fiber deficient diets, where resident polysaccharide degrading bacteria begin to utilize the mucus layer of the intestine, which can reduce intestinal barrier function leaving the host increasingly vulnerable to pathogen invasion (54). Feeding highly digestible low fermentable wheat based diets to pigs increases abundance of *Akkermansia*, a microbe known to utilize host-glycans, emphasizing the adaptability of the microbiota to utilize host substrates when dietary fiber is scarce (49).

### Maternal Fiber Supplementation on Progeny Microbiota

In natural settings offspring of monogastrics derive their gut-associated microbiota through vertical transmission during the birthing or hatching process. The minimal distance between the digestive tract and birthing canal is likely no evolutionary coincidence. In commercial swine production piglets fecal microbiota first resembles that of the environment (floor, sow milk, and sow nipple); however, soon reflects that of the sow, emphasizing the importance of the sow microbial composition (55). Although hens externalize eggs through their vent, a common external opening for excretion of fecal matter, the practice of cleaning eggs pre-hatch removes many co-evolved avian microbes leaving newly hatched chicks to colonize with environmentally derived non-host-adapted microbiota.

Due to the fact piglets receive their colonizing microbiota from the sow (55), beneficial manipulation of sow microbiota with dietary fiber may directly influence the intestinal microbiota of her piglets. The concept of fetal programming through maternal nutrition is not new, and it has been shown that maternal seaweed extract supplementation can reduce both sow fecal *Enterobacteriaceae* populations at parturition and piglet *E. coli* populations at weaning (56). Both wheat bran and inulin supplementation of sows during gestation and lactation have shown to impact piglet microbiota and fermentation profiles (57) with inulin also able to reduce enterobacteria (58). Although fiber supplementation of sow diets has shown to impact piglet microbial profiles, the changes observed may be more related to altered colostrum and milk composition rather than maternal microbial changes. After parturition there is a 1–3 week period whereby piglets rely exclusively on the sow for nutrition and research in humans has demonstrated the importance of milk composition in shaping the neonatal intestinal microbiota (59). In particular, the composition of milk oligosaccharides is of great interest, as these heterogeneous mix of soluble glycans are indigestible by the host but provide a fermentative substrate for the colonizing intestinal microbiota (60, 61).

Sows also produce milk oligosaccharides that are fermentable by piglet microbiota (62), which suggest they play a key role in colonizing microbiota composition (63). Current literature suggests dietary supplementation of sows with short-chain fructo-oligosaccharides (scFOS) during nursing can increase microbial fermentative capacity in their suckling piglets, stimulating the development of intestinal immune defenses including increased ileal cytokine secretions, mucin secreting goblet cell numbers, and improved vaccine-specific IgA levels (64). Increased fermentative capacity in piglets suckling from scFOS supplemented sows may be from altered porcine milk oligosaccharide composition, as recent literature has suggested that supplementing nursing sows with chitooligosaccharides (COSs) significantly alters milk oligosaccharide composition (65). The effects of supplementing sows with soluble fiber (pregelatinized waxy maize starch and guar gum) can also be immediately recognized by the improved piglet growth rates and associated increase in plasma growth hormone, insulin-like growth factor-1, and reduced incidence of diarrhea (66). In the study by Cheng et al. (66), piglets suckling from soluble fiber supplemented sows also had remarkable changes in their microbial composition, with increased relative abundance of *Bacteroides, Lactobacillus, Roseburia, Fusobacterium*, and *Acinetobacter* that was accompanied by improvements in markers of intestinal integrity (plasma zonulin, endotoxin, and diamine oxidase). Maternal fiber supplementation can also affect other colostrum and milk components essential for piglet immune development. Sows supplemented with scFOS have shown to have increased colostral IgA and transforming growth factor beta-1 which subsequently supported piglet mucosal immune development by increasing secretory IgA production in Peyer's patches and activated T cells (67). This emphasizes the important and often overlooked concept of maternal nutritional programming on offspring microbial and immune development.

## Immune Programming With SCFA

It is well-accepted that the gut-associated microbiota have co-evolved with their respective host and play a vital role in immune maturation and function and protection against pathogens (68, 69). The relationship between gut microbiota and immune development is exemplified in germ-free animal models, which have defective immune systems whereby colonization with live microbial communities recapitulates immune development and function (70). Uncovering the mechanisms of how microbial communities benefit host immune function is in its infancy; however, appear highly connected to microbial fermentation metabolites, SCFAs. The production of SCFAs, particularly butyrate, can enhance intestinal epithelial cell barrier function, the first line of defense against invading pathogens (71) and helps maintain this physical barrier by stimulating goblet cell differentiation and mucus production (72). Short chain fatty acids promote the differentiation and function of colonic regulatory T cells, which maintain gut homeostasis by inhibiting effector T-cell function and increasing IL-10 production, important in preventing excessive inflammation (73, 74). The presence of specific nonpathogenic bacteria, such as *Bacteroides thetaiotamicron*, can also inhibit host inflammatory responses by promoting the nuclear exportation of NF-κB, a transcription factor that triggers proinflammatory gene expression (75). Although intestinal inflammation may sometimes be necessary to clear intestinal pathogens, restoring intestinal homeostasis as quickly as possible is necessary to maintain animal health and performance.

## Maintaining an Anaerobic Environment With SCFA

**Figure 3:**
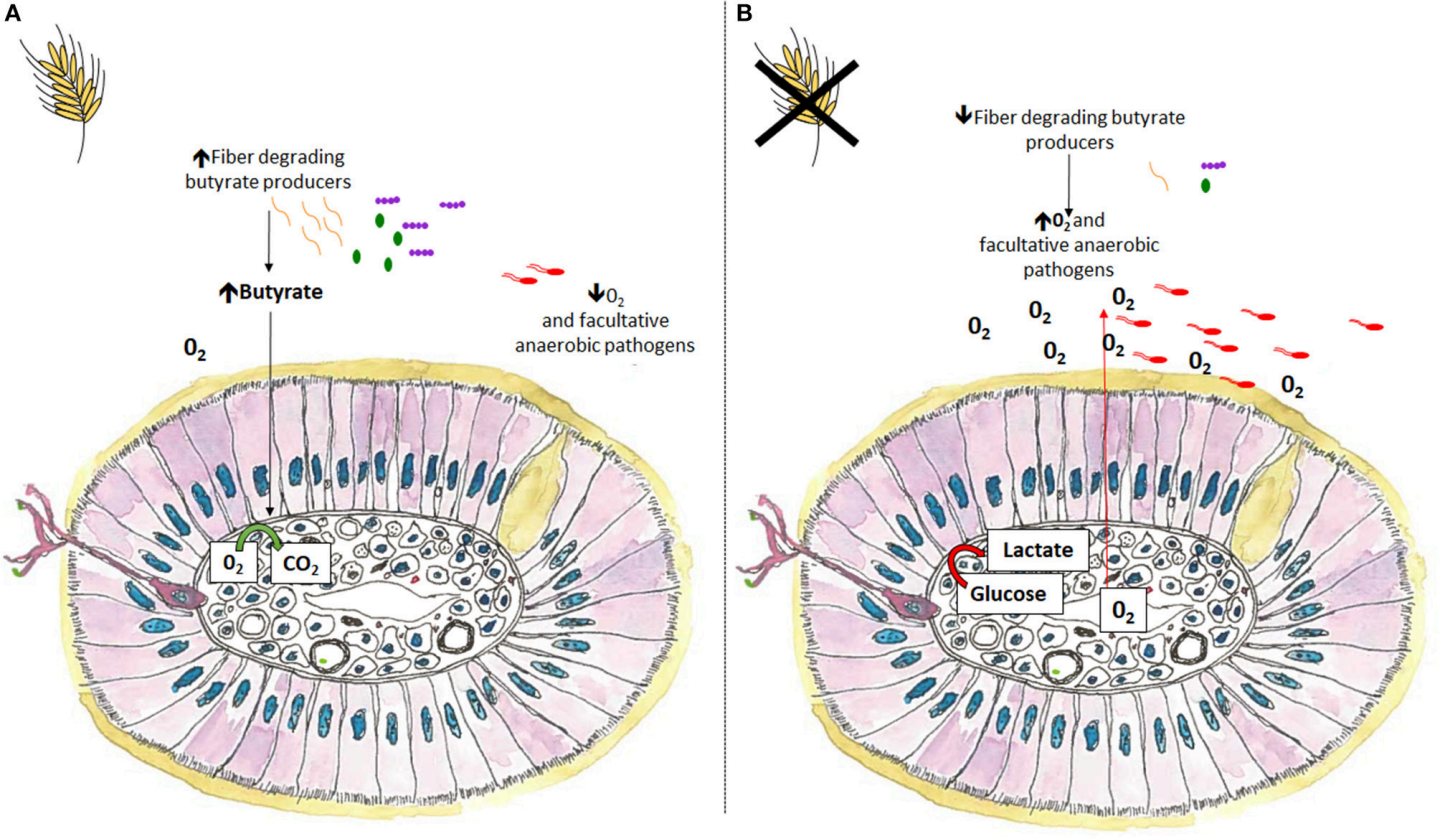
A transverse cross section of colonic villi in the presence or absence of dietary fiber. **(A)** Inclusion of dietary fiber helps maintain intestinal homeostasis and improves disease resilience by maintaining a hypoxic environment. Dietary fiber facilitates the expansion of anaerobic butyrate producers, which subsequently increases butyrate concentrations, reducing luminal oxygen, and limiting the expansion of facultative anaerobic pathogens. **(B)** Alternatively, in the absence of dietary fiber facultative anaerobic pathogens, including certain *E. coli* and *Salmonella* species may expand at the expense of oxygen sensitive butyrate producers. In the absence of butyrate, enterocytes use anaerobic glycolysis to obtain energy, a process that increases epithelial oxygen concentrations creating a favorable niche for facultative pathogens such as *Salmonella* to flourish.

The fermentation metabolite butyrate is used preferentially as an energy substrate by intestinal epithelial cells and plays a major role in maintaining homeostasis by keeping the intestine anaerobic. During microbial colonization the GIT goes from being aerobic to anaerobic. In a homeostatic state the intestine remains anaerobic with anaerobic bacteria outcompeting aerobes and facultative anaerobes. During dysbiosis facultative anaerobic Proteobacteria, such as *E. coli* and *Salmonella*, characteristically expand at the expense of oxygen sensitive butyrate producers, disrupting the anaerobic intestinal environment (76). Referring to dysbiosis as “dysanaerobiosis” elegantly summaries the change in intestinal environment from hypoxic to micro-aerophilic and the subsequent shift from obligate anaerobes to facultative anaerobes (77). Inclusion of dietary fiber may help prevent or ameliorate the micro-aerophilic environment that occurs during dysbiosis by providing a fermentative substrate to anaerobic butyrate-producing bacteria (Figure 3). In a homeostatic environment host intestinal tissues use butyrate as an energy substrate via β-oxidation, a process that consumes considerable amounts of oxygen helping to maintain an anaerobic environment (76, 78). In the absence of butyrate, enterocytes use anaerobic glycolysis to obtain energy, a process that increases epithelial oxygen concentrations creating a favorable niche for facultative pathogens such as *Salmonella* to flourish (76, 79). To maintain and improve piglet and poultry gut health, nutritional strategies should aim at restoring the hypoxic intestinal environment through the expansion of butyrate producers to prevent facultative anaerobic expansion.

## Dietary Fiber on Intestinal Disorders/Diseases

Inclusion of dietary fiber can support colonization of beneficial commensal microbiota that competitively exclude pathogens, enhance maturation, and barrier function of the GIT through metabolite production, and directly block adhesion of pathogenic microbes to the intestinal epithelium by providing alternative adhesion sites (80). One of the most common causes of reduced animal performance and economic loss in swine production is the incidence of post-weaning diarrhea caused by opportunistic pathogens such as *E. coli* and *Salmonella*. Historically highly digestible low fiber diets have been used for newly weaned pigs in efforts to improve digestibility and animal performance. However, it has since been proposed that there is likely at least a minimum dietary fiber requirement for piglets to achieve optimal gut health (81). As such, inclusion of insoluble non-starch polysaccharides (iNSP) such as oat hulls have shown to reduce diarrhea incidence in piglets (81, 82). Although oat hulls are highly insoluble and lignified in nature, they are also able to reduce fecal biogenic amines, cadaverine, and β-phenylethylamine, from protein fermentation, signifying oat hulls can beneficially influence dietary fermentation patterns (82). Inclusion of 40 g/kg of wheat bran in piglet diets, another dietary source of iNSP, can also reduce intestinal enterobacteria populations and increase butyric acid concentrations in young piglets, further suggesting the ability of piglet gut microbes to utilize insoluble fiber and provide protection (83). Additionally, when challenged with *E. coli* K88, piglets supplemented with coarsely ground wheat bran had reduced diarrhea severity, increased SCFA concentrations (84), and reduced ileal *E. coli* K88 adhesion (85).

There is conflicting evidence as to whether or not inclusion of soluble fiber is detrimental or beneficial to disease resistance in piglets and has been reviewed previously (80, 86). An older literature has reported that increasing dietary soluble non-starch polysaccharides (sNSP) from 1 to 6% can increase haemolytic *E. coli* in the small intestine from 1.3 × 10^4^ to 8.0 × 10^9^ (87). Although increasing levels of dietary sNSP can increase SCFA concentrations, digesta viscosity is also linearly related with sNSP intake and is suggested be the cause of intestinal *E. coli* proliferation (88). However, more recently sNSP have shown to be protective against post-weaning diarrhea, likely through the promotion of commensal microbiota proliferation, SCFA production, and subsequent maintenance of an anaerobic environment. Inclusion of 50–150 g/kg of inulin was shown to increase the *Lactobacillus*:coliform ratio and SCFA concentrations (89) while reducing the occurrence of diarrhea when challenged with *E. coli* (89, 90). Enrichment of commensal microbiota such as *Lactobacillus* with sNSP (91) may induce growth inhibition or competitive exclusion to *E. coli* (92).

As discussed above another mechanism by which DF may reduce diarrhea incidence and pathogen colonization is by improving intestinal barrier function. It has been shown that inclusion of 10% wheat bran fiber or pea fiber into piglet diets can improve intestinal barrier function (increased villous height: crypt depth ratio, colonic goblet cells, and peptide trefoil factors) potentially mediated through changes in microbial composition, namely increases in *Lactobacillus* and *Bifidobacterium* populations (20). Furthermore, wheat bran fiber and pea fiber were observed to reduce diarrhea incidence in comparison to maize fiber and soybean fiber (20), suggesting source, compositional and functional characteristics of fiber are important factors to take into consideration. There is also evidence that fermentable fiber can benefit pre-weaned pigs, where piglets fed milk replacer supplemented with 7.5 g/L of either FOS or soy polysaccharides vs. methylcellulose can increase SCFAs in the colon, improve intestinal function (increased glutamine transport), and can inhibit *Salmonella* induced diarrhea (93).

Swine dysentery (SD) is another common contagious diarrheal disease observed in the grower-finisher phase of swine production caused by the intestinal spirochaete *Brachyspira hyodysenteriae*. Recent work has shown that diets high in fructans and galactans from chicory root and sweet lupins can protect pigs from infectious SD (94, 95), which may be due to increased abundance of commensal microbiota, *Bifidobacterium thermacidophilum* subsp. *porcinum* and *Megasphaera elsdenii*, lactate producers and lactate utilizing butyrate producers, respectively (95). More recent research also observed that although lupins can delay the onset of disease, 80 g/kg inclusion of inulin can reduce the risk of developing SD (96). A study by Hansen et al. (97) also confirmed that increasing dietary inulin from 0 to 80 g/kg reduces the risk of pigs developing SD when challenged directly with *Brachyspira hyodysenteriae* and the protective effect was accompanied by a linear increase in cecal SCFAs and reduction in protein fermentation metabolites. It is hypothesized that inulin acts by modifying microbial fermentation patterns, potentially reducing the protein:carbohydrate ratio in the hindgut increasing carbohydrate fermentation while suppressing protein fermentation, thereby inhibiting SD colonization (97).

A severe intestinal disorder in poultry is necrotic enteritis and is caused by the pathogen *C. perfringens*. Feeding whole wheat has been shown reduce and *C. perfringens*, the causal pathogen of necrotic enteritis (98, 99). It is suggested by authors that whole wheat improves gut health of chickens by reducing gizzard pH, increasing retention time and viscosity creating an inhospitable environment for pathogen survival into the lower intestinal tract (98). Acetylated resistant starch has also been shown to improve gut health and reduce severity of a *C. perfringens* challenge through reducing luminal pH through specific SCFA delivery (100).

Controlling *Salmonella* colonization in poultry flocks is another global priority to reduce potential zoonotic contamination of meat products. A 1% inclusion of wheat bran with a reduced particle size (280 μm) into broiler diets was able to reduce levels of cecal *Salmonella* colonization (1.3 vs. 3.6 Log CFU/g in control) and *Salmonella* shedding post-challenge. *In vitro* fermentation of 280 μm wheat bran resulted in increased production of butyrate and propionate compared with larger particle sizes (101). Inclusion of whole wheat in broiler diets has also shown to increase gizzard fermentation reducing gizzard pH and subsequent *Salmonella* Typhimurium post-challenge; further suggesting feed structure and particle size can influence pathogen colonization (99). Incubating *Salmonella* with wheat bran (280 μm) fermentation products can reduce hilA expression, a transcriptional activator of *Salmonella* pathogenicity island I vital for *Salmonella's* entry into epithelial cells (102). A component of wheat bran, arabino-xylooligosaccharides, can also reduce *Salmonella* colonization of the cecum and subsequent *Salmonella* shedding (103). Other fiber types including FOS and mannan-ologisaccharides have shown to inhibit the growth and colonization of *Salmonella in vitro* (104) and *in vivo* (105). Although there is much evidence to suggest supplementing dietary fiber to pigs and poultry is beneficial to gut health and disease resistance, research needs to focus on defining the mechanisms of action to help develop optimal nutritional strategies to further improve animal health. It must be recognized that there are likely numerous nutritional strategies that utilize dietary fiber to improve gut health of pigs and poultry depending on environment, health status, life stage, and feeding objective (growth vs. longevity).

## Conclusion

Although dietary fiber was recognized as an anti-nutritional factor in the past, there is increasing interest in its inclusion in monogastric animal's diets due to potential functional benefits to the host, primarily on the intestinal health. The benefits are primarily due to fermentation of DF in the distal GIT. The fermentation metabolites and interaction of DF with the intestinal environment affect the intestinal histomorphology, mucosa, microbial community, and immune system, altogether named as “intestinal health.” Based on the available information, it can be concluded that inclusion of dietary fiber can be a strategy to improve gut health, thereby overall health and production of monogastric animals in the post-antibiotic era. However, type, form, physico-chemical properties as well as the amount of DF inclusion in diets need to be considered strategically as there is wide variation in their composition and subsequently their effects on intestinal health of monogastric animals.

## Author Contributions

JF, UT, and LL wrote this review manuscript. BW reviewed the manuscript and provided critical suggestions and comments. RJ decided on the review topic, reviewed the literature and manuscript, and provided critical suggestions and comments.

### Conflict of Interest Statement

The authors declare that the research was conducted in the absence of any commercial or financial relationships that could be construed as a potential conflict of interest.
